# Efficient and Stable Hydrogen Evolution from HI Splitting Using a Robust 2D Tin-Iodide Perovskite

**DOI:** 10.1021/acs.jpcc.5c07925

**Published:** 2026-02-02

**Authors:** Samiksha Mukesh Jain, Samrat Das Adhikari, Camilo A. Mesa, Hind Benzidi, José Manuel González-Acosta, Andrés F. Gualdrón-Reyes, Núria López, Sixto Giménez, Iván Mora-Seró

**Affiliations:** 1 Institute of Advanced Materials, Universitat Jaume I, Avinguda de Vicent Sos Baynat, s/n, Castelló de la Plana 12006, Spain; 2 Institute of Physical Chemistry, Polish Academy of Sciences, Warsaw 01-224, Poland; 4 Institute of Chemical Research of Catalonia (ICIQ-CERCA), The Barcelona Institute of Science and Technology (BIST), Av. Països Catalans 16, Tarragona 43007, Spain; 5 Department of Physical and Inorganic Chemistry, University Rovira I Virgili, Marcel·lí Domingo s/n, Tarragona 43007, Spain; 6 Facultad de Ciencias, Instituto de Ciencias Químicas, Isla Teja, Universidad Austral de Chile, Valdivia 5090000, Chile

## Abstract

Photocatalytic hydrogen (H_2_) production with 2D Ruddlesden–Popper tin-iodide perovskites has recently emerged as a promising route toward sustainable solar-to-fuel conversion. However, a major limitation of these systems lies in their rapid degradation caused by tin and iodide oxidation. In the present study, we report the synthesis of 4-fluorophenethylammonium tin-iodide (4FPSI) perovskite microcrystals in a mixture of hydroiodic acid (HI) and H_2_O, which exhibit remarkable long-term photostability and sustained photocatalytic H_2_ production via HI splitting. Intermittent light irradiation was shown to further boost H_2_ production by promoting efficient charge separation and suppressing the accumulation of trapped charge carriers that drive recombination. Notably, reused and aged materials showed enhanced photocatalytic performance, which theoretical simulations attributed to surface reconstruction that exposes additional tin catalytic active sites. The samples that underwent degradation after multiple photocatalytic tests could be recovered through a simple chemical treatment and restore the H_2_ production capability. Together, these findings highlight tin-iodide perovskites as highly promising photocatalysts for solar H_2_ production, combining durability, recyclability, and facile recovery strategies to simultaneously advance all key performance metrics.

## Graphical abstract

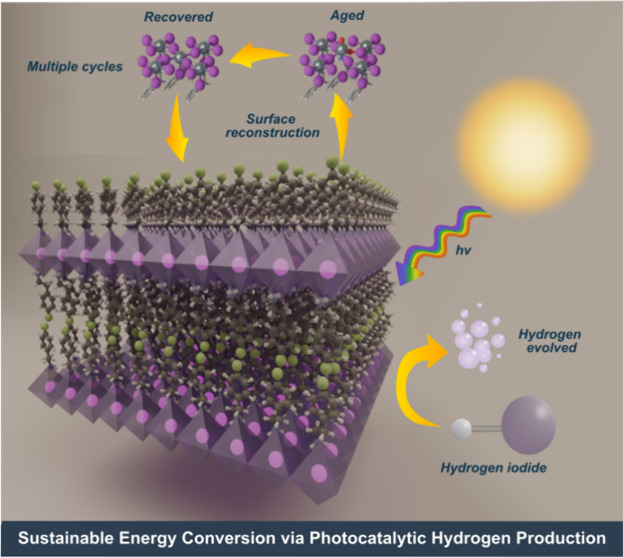

## Introduction

Halide perovskites have revolutionized the field of optoelectronics due to their outstanding properties, including high optical absorption, tunable bandgap, and exceptional charge-carrier dynamics.^1,2^ Among halide perovskites, lead-based systems exhibit the highest performance,^3–5^ but their toxicity and potential for bioaccumulation of lead raise serious environmental and health concerns, underscoring the need for safer alternatives. Tin halide perovskites exhibit similar optoelectronic behavior, adding to environmental friendliness, and high potential for green energy generation.^6,7^ Halide perovskites are classified according to their bulk structural dimensionality, such as 0D, 2D, and 3D, while their typical formulas in the case of tin perovskites are A_2_Sn­(IV)­X_6_, A_2_Sn­(II)­X_4_, and ASn­(II)­X_3_, respectively, where A accounts for the organic cation in the structure.^8^ In particular, FASnI_3_ (FA: formamidinium), a 3D tin-iodide perovskite with a narrow direct bandgap, has demonstrated excellent photovoltaic performance in solar cells.^9–11^ However, a major challenge for tin-based perovskites is their pronounced instability in the presence of moisture, which accelerates the oxidation of Sn^2+^ to Sn^4+^ and leads to rapid material degradation.^12^ Breakthroughs of the moisture stability of lead-based 3D perovskites have been achieved by structural engineering. This includes slicing the corner-shared octahedra anionic layers and replacing the A-site cation with a long-chain organic ammonium cation positioned between the inorganic layers, forming 2D-layered halide perovskites (2DLHP) known as Ruddlesden–Popper perovskites. These materials have demonstrated extended moisture stability, making them considerably more effective catalysts than their 3D components.^5,13–15^ Inspired by these advances, similar strategies are now being adapted to improve the environmental stability of tin-based perovskites.^16,17^

2DLHPs based on organic ammonium tin-iodide perovskites exhibit strong optical absorption in the orange-red region, making them highly promising for solar-driven hydrogen (H_2_) generation. Yet, their application in photocatalytic applications, particularly in photocatalytic water splitting, which remains largely unexplored. To date, 3D tin halide perovskites have shown extreme water instability, undergoing rapid degradation upon contact with water. A comparison of water stability between MASnI_3_ and DMASnBr_3_ (DMA = dimethylammonium) following ab initio molecular dynamics simulation revealed that the SnI_2_-terminated MASnI_3_ surface undergoes faster dissolution upon water contact. On the other hand, DMASnBr_3_ is terminated with a disconnected 0D SnBr_3_ layer, which is amorphous in nature and hence the stronger Sn–Br bond protects from water-induced dissolution.^18^ In a report by Malavasi and co-workers, the water stability of DMASnBr_3_ has been leveraged for photocatalytic H_2_ production.^19^ While DMASnBr_3_ demonstrated promising stability, its iodide analogue exhibited water-induced reversible bandgap modulation. Upon exposure to water, the material changed color from black to yellow, corresponding to a bandgap shift from 1.32 eV (dry state) to 2.48 eV (hydrated state).^20^ To date, no photocatalytic performance has been reported for water-dispersed tin-iodide perovskites with optical stability.

In contrast, H_2_ production via hydroiodic acid (HI) splitting is emerging as a promising strategy in comparison to water splitting, offering lower energy requirements. This is in contrast due to its two-electron mechanism (compared to the four electron process for water splitting) and a significantly lower oxidation potential (0.535 V for I^–^/I_2_, vs 1.23 V for H_2_O/O_2_), making the process thermodynamically more favorable.^3,21,22^ Tin-iodide perovskites are particularly well-suited as microcrystalline photocatalysts for solar-driven H_2_ generation via HI splitting. Their synthesis typically involves HI as the iodide source, which can also act as a sacrificial agent during the reaction. When dispersed in solution, these microcrystals interact with the excess iodide anions, establishing a dynamic equilibrium between iodide in the perovskite and the surrounding solution. This mechanism, previously demonstrated with MAPbI_3_, has been shown to enhance perovskite stability and enhance photocatalytic H_2_ evolution.^3^ A similar behavior is expected for the 2DLHPs employed in the present study, as shown below.

In this work, we report the synthesis of the 4-fluorophenethylammonium tin-iodide (4FPSI) perovskite, which exhibits remarkable long-term stability for more than 1 year in aqueous HI solution. The photocatalytic H_2_ production was evaluated by using HI as both the synthesis medium and the sacrificial agent. Notably higher H_2_ production was observed under intermittent illumination compared to that under continuous light irradiation. The material also exhibited improved performance upon aging, with repeated use of the same sample yielding enhanced activity. Importantly, all the photocatalytic tests were carried out without any co-catalysts. The 4FPSI sample aged for 9 months achieved a H_2_ production rate of 30.8 μmol.g^–1^.h^–1^, representing, to the best of our knowledge, the highest reported value for a co-catalyst-free tin-based perovskite to date. Furthermore, the degraded material could be successfully regenerated through a simple solution-based recrystallization process.

## Materials and Methods

### Materials

Hydroiodic acid (HI; 57 wt % in H_2_O, distilled, stabilized, 99.95%) and hypophosphorous acid (H_3_PO_2_; 50 wt % in H_2_O) were purchased from Sigma-Aldrich. Tin­(II) oxide (SnO; 99%) was purchased from Alfa Aesar, and 4-fluorophenethylamine (4FPEA) was purchased from TCI. All materials were used as received with no further purification.

### Synthesis of 4FPSI

4FPSI was synthesized by combining 1.5 mL of deionized water, 1.5 mL of HI, and 0.3 mL of H_3_PO_2_ in a three-neck round-bottom flask under an inert nitrogen atmosphere. The solution was kept at room temperature until it turned colorless, confirming the stabilization of HI in the presence of H_3_PO_2_. Next, 134 mg of SnO was added to the flask, and the temperature was increased to 100 °C. The solution was stirred for 30 min until the precipitate dissolved and the solution turned yellow. Then, 0.26 mL of 4FPEA was added. Once the precipitation started, the mixture was cooled in an ice bath for 2 min. The solution was centrifuged at 6500 rpm for 5 min, and the filtrate was collected in a separate vial. The powdered product was washed with hexane three times using vacuum suction filtration. Finally, the powder was resubmerged in the filtrate and sonicated for 30 min to improve dispersion.

### Recovery of 4FPSI

The degraded perovskite (100 mg) was taken in a beaker, and 10 mL of the solution contained 4.5 mL of HI (57%), 1.5 mL of H_3_PO_2_ (50%), and 4 mL of DI water. Then, the mixture was heated to 150 °C for 15 min to evaporate the water and densify the solution. Once the total volume of the solution was reduced to nearly 50%, the solution was placed in an ice bath for 30 min. Finally, the product was collected from the same solution.

For the recrystallization of 4FPSI using HCl+HI, 2 mL of HCl and 2 mL of DI water were used instead of 4 mL of DI water. The rest of the procedure was identical to the above.

### Characterization

SEM images were taken with a field emission scanning electron microscope (FEG-SEM, JEOL 3100F) operated at 15 kV.

XRD measurements were performed using an X-ray diffractometer (D8 Advance, Bruker-AXS) (Cu Kα, wavelength λ = 1.5406 Å), with a tube voltage and intensity of 40 kV and 40 mA, respectively, between the Bragg angle range of 4–70°, and a step size of 0.05°. The detector was a BRUKER-binary V3, using a scan range from 4.0 to 70.0° (2θ °) and a scan step size of 0.05° (2θ °). Measurements were registered at room temperature (298 K).

Photoluminescent (PL) spectra were measured by taking 50 mg of the washed sample and redispersing it in 3 mL of HI+H_2_O solution. This solution was then sonicated for 10 min for a better dispersion. The spectra were measured with a HORIBA Fluorolog spectrofluorometer, under the wavelength range of 550–800 nm.

The UV–vis absorption spectrum was acquired with a spectrophotometer (Varian Cary 300 BIO) in the wavelength range of 250–650 nm.

### Photocatalysis

Perovskite microcrystals (200 mg) were dispersed in 4.5 mL of HI (57%), 1.5 mL of H_3_PO_2_ (50%), and 4 mL of DI water in a 25 mL capped vial. The solution was sonicated for 10 min. The same dispersion was transferred to a three-necked quartz flask, capped with rubber septa. The dispersion was purged with Ar for 40 min before the measurements. The H_2_ generation was quantified by gas chromatography measurements, coupling the sealed three-necked quartz flask containing the perovskite solution to an AGILENT MicroGC 490 gas chromatograph. The outlet gas was analyzed every 5 min. A thermal conductivity detector, μTCD together with a narrow-bore column, was used. Throughout the measurement, the solution was continuously stirred to ensure that the material was well dispersed. The light source used was a 300 W Xe lamp with an AMG 1.5. The light intensity was adjusted to 100 mW cm^–2^ using a Si photodiode.

### Reuse of 4FPSI

The solution containing the perovskite microcrystals in HI, H_3_PO_2_, and deionized water used for hydrogen evolution measurements was stored in a sealed vial under dark ambient conditions after each experiment. The same solution was subsequently reused for additional hydrogen production tests to investigate the effect of aging.

## Results and Discussion

### Structural and Optical Characterizations of 4FPSI Perovskites

The conventional synthesis of 4FPSI perovskite microcrystals (MCs) typically uses 57% HI as both the iodide source and solvent.^23,24^ However, the use of excess iodide leads to severe oxidation of the MCs, evidenced by a rapid color change of the solution, from faint orange to black-red within 2 h. This oxidation not only accelerates the degradation of tin-iodide perovskites but also results in substantial HI waste.^25^ To address these issues, we diluted 57% HI with H_2_O, effectively reducing iodide oxidation and minimizing HI consumption. The optimal HI concentration to dissolve SnO (without forming hydrolyzed tin products) was determined as a 50/50 mixture of H_2_O and 57% HI. In the synthesis process, SnO was first reacted with HI and H_3_PO_2_ to form a clear solution of tin-iodide complexes. Subsequently, 4-fluorophenethylamine was added to induce the crystallization of 4FPSI MCs, as illustrated schematically in Figure 1a. We have previously reported the use of SnO as a low-cost alternative to SnI_2_ for the synthesis of Sn-perovskite MCs, following an alternative method for the fabrication of Sn-perovskite LEDs with enhanced performance.^26^

**Figure 1:**
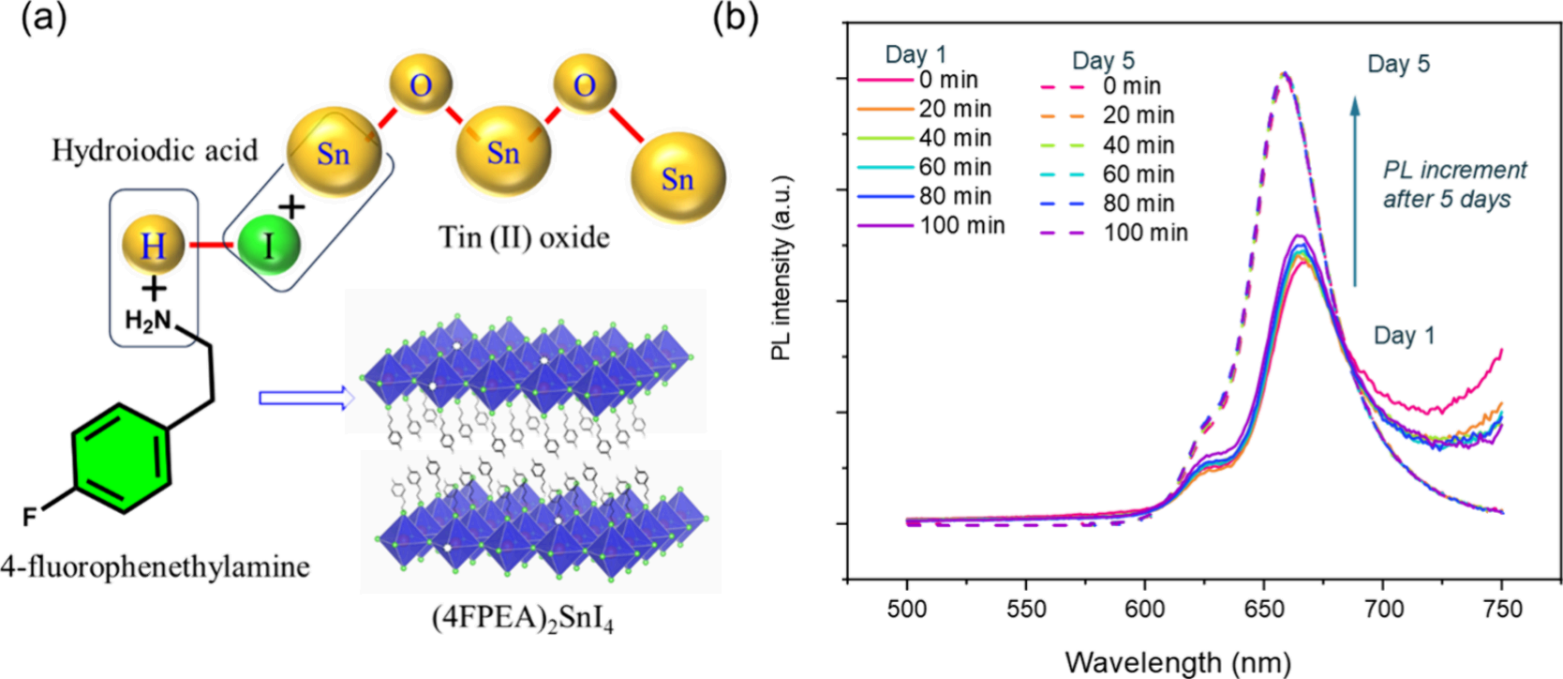
(a) Scheme of the reaction mechanism leading to 4FPSI perovskite MCs in HI/H_2_O, and (b) PL spectra of the MCs measured at different times.

The photoluminescence (PL) spectra of the fresh 4FPSI MCs dispersed in an HI/H_2_O solution showed a slight increase in PL intensity during the first 100 min, see Figure 1b, demonstrating good photostability of 4FPSI MCs. Interestingly, after 5 days, the same sample exhibited an increased PL intensity, compared to the freshly prepared material. This optical enhancement could be attributed to a halide deficiency recovery mechanism, similar to that reported for Pb-based perovskite nanocrystals, where postsynthesis halide treatment restores surface halides and enhances PL intensity.^27^ Notably, no triiodide formation was detected by ultraviolet–visible (UV–vis) absorption of the supernatant solution, indicating that the aged solution does not promote iodide oxidation, see Figure S1a. The UV–vis diffuse reflectance spectrum of the fresh 4FPSI MCs is provided in Figure S1b. Furthermore, phase stability was preserved in the material aged for 5 days, suggesting that Sn^2+^ oxidation was effectively suppressed, and the perovskite structure remained intact. Complementary DFT point defect formation energy simulations under poor-, medium-, and rich-halide conditions, see Figures S2 and S3, reveal that tin vacancies, V_Sn_, and iodine interstitials, I_i_, are energetically favorable in iodine-rich environments. These defects are known to introduce deep and shallow trap states, respectively, which can serve as nonradiative recombination centers. The observed PL enhancement after 5 days suggests that these defects are progressively passivated, potentially through environmental equilibrium, common-ion suppression, or intrinsic self-healing processes. In contrast, under iodine-poor conditions, tin interstitials, Sn_i_, become the dominant low-energy defects while the formation energy of V_Sn_ increases significantly. These Sn-related point defects may contribute to increased electron compensation or additional trap states, which would typically suppress PL.

### Photocatalytic HI Splitting

Photocatalytic H_2_ production via HI splitting was investigated using the 4FPSI perovskite without the addition of any cocatalysts. The fresh material was dispersed in an aqueous HI solution, and the photocatalytic activity was evaluated as described in the Materials and Methods section. Upon 5 h of continuous photoirradiation, the photogenerated electrons reduced H^+^ to molecular H_2_, see the red curve in Figure 2a. The corresponding average H_2_ production rate was 2.6 μmol·g^–1^·h^–1^, see Figure 2b. However, the H_2_ evolution decreased over time upon continuous irradiation, probably due to increased charge recombination, reducing the number of available charge carriers. Interestingly, when intermittent irradiation was employed, the light/dark cycling strategy led to enhanced H_2_ production, see blue curve in Figure 2a. For intermittent irradiation, samples were exposed to light for 60 min, followed by 15 min in the dark, during which the reaction vessel was covered with an aluminum foil to further avoid stray light exposure. During the dark intervals, no H_2_ evolution was observed, confirming the photocatalytic nature of the process, see Figure 2a. Six hours of intermittent irradiation (5 h illuminated +1 h dark), see Figure 2a, led to a higher average H_2_ production rate of 4.2 μmol·g^–1^·h^–1^, see Figure 2b, outperforming the continuous operation mode. This observation suggests several advantages of intermittent illumination: (i) to minimize unnecessary energy input, (ii) to decrease catalyst degradation, and (iii) to enhance the overall H_2_ production efficiency. Further, time-resolved photophysical measurements (e.g., transient or time-resolved PL) could provide direct insight into charge-carrier dynamics under intermittent illumination and help elucidate the underlying mechanism; however, such quantitative analyses are beyond the scope of the present study and will be pursued in future work.

**Figure 2:**
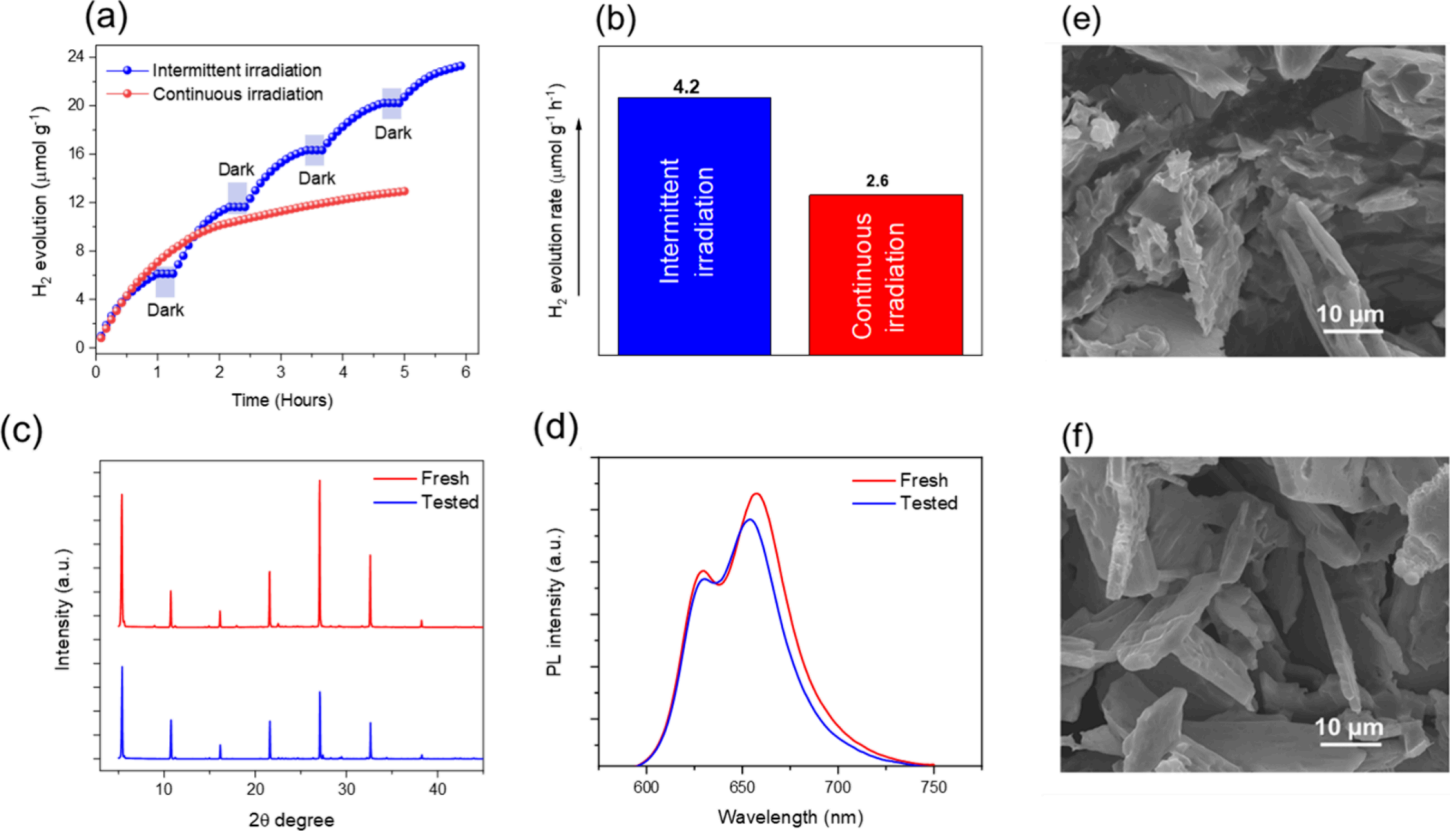
(a) Photocatalytic H_2_ production under continuous irradiation (red curve) and intermittent irradiation (blue curve), and (b) corresponding H_2_ production rate. Characterization of the fresh and the tested samples: (c) XRD patterns and (d) PL spectra. SEM images of (e) fresh and (f) tested samples presenting an identical morphology.

While the decreased average H_2_ evolution rate under continuous illumination suggested possible photocatalyst degradation, a thorough structural and morphological analysis revealed no significant signs of material deterioration. After the 5 h photocatalytic test, shown in Figure 2a, the samples retain their original color, X-ray diffraction (XRD), see Figure 2c, and PL features, see Figure 2d, indicating high stability of the 4FPSI perovskite during photocatalysis. In addition, the platelet-type morphology of fresh 4FPSI was preserved, as shown in the scanning electron microscopy (SEM) comparing fresh and tested samples, see Figures 2e and 2f, respectively. The fresh sample presents an average thickness of 1.71–2.11 μm, while the tested sample displayed no noticeable morphological changes with thickness in the same range, 1.76–2.06 μm, further confirming the structural robustness of the perovskite after photoirradiation (see Figure S4).

Previous reports on photocatalytic HI splitting^3,28^ suggest that photogenerated holes at the 4FPSI perovskite surface can oxidize iodide ions (I^–^) to iodine (I_2_), which typically react with excess iodide in solution to form triiodide (I_3_^–^). Surprisingly, in our case, no evidence of triiodide formation was detected in the photoirradiated 4FPSI samples, see Figure S1, which contrasts with previous studies on MAPbI_3_ perovskites, where photocatalytic HI splitting resulted in clear triiodide formation.^3,28^ This behavior may arise from the presence of hypophosphorous acid (H_3_PO_2_), which acts as a strong reducing agent capable of rapidly converting I_2_ or I_3_^–^ back to I^–^, thereby suppressing triiodide accumulation during photocatalysis.^29,30^

To explore the effect of aging and reusing on the photocatalytic activity, the same 4FPSI sample was tested for H_2_ production over multiple cycles without the addition of any other chemical (reused samples), see Figure 3. The samples were stored in sealed vials under ambient conditions in the dark. Remarkably, after aging for 4 days, the reused sample exhibited a pronounced enhancement in performance, see Figure 3a, followed by a gradual decline over more extended periods, see H_2_ evolution for 5–7 days in Figure 3a,b. The H_2_ production of the fresh sample was re-evaluated after 9 months of storage (in a sealed vial under ambient conditions in the dark), and again after an additional 3 months, i.e., 12 months since the initial measurement, see Figure 3c. The fresh sample showed the expected performance, while the reused samples showed superior performance, see Figure 3d. Notably, the enhancement in H_2_ evolution does not increase monotonically with aging time. The average H_2_ production rate of the sample reused after 9 months was ∼30.8 μmol·g^–1^·h^–1^, while that for the same sample tested after 12 months was ∼9.20 μmol·g^–1^·h^–1^, see Figure 3d. The external quantum yield (EQE) of the samples tested is summarized in Table 1. While the present study focuses on the general benefits of intermittent illumination and the aging effect of the 4FPSI photocatalyst, further developments should include targeted optimization of these materials for either indoor or outdoor photocatalytic operation.

**Figure 3:**
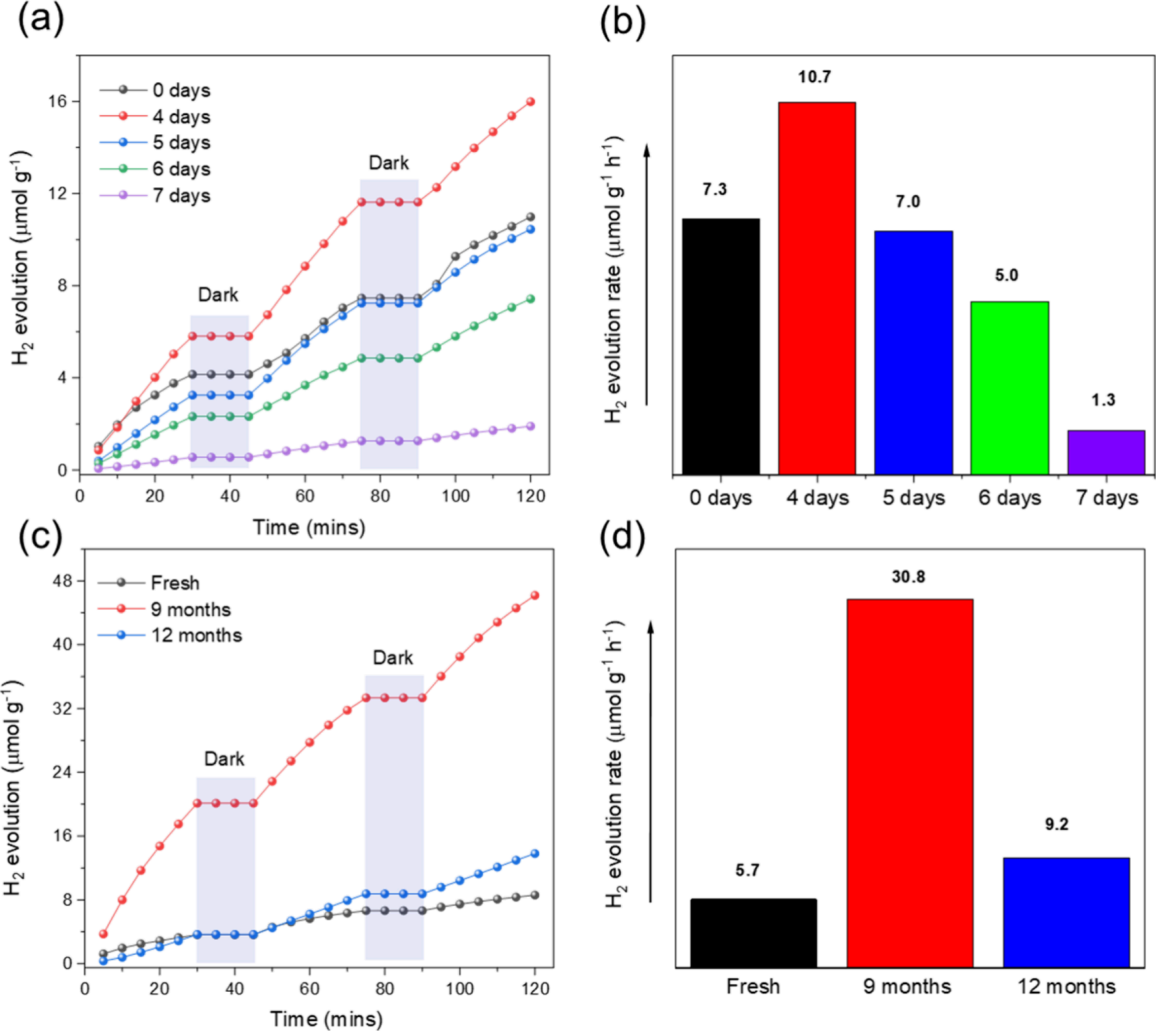
(a) Photocatalytic H_2_ production of the samples tested several times, and (b) their corresponding average H_2_ evolution rate. (c) Photocatalytic H_2_ production for the fresh and reused samples, and (d) their corresponding average H_2_ evolution rate. The fresh sample was tested and then reused after 9 months and 1 year, respectively.

**Table 1:** External Quantum Efficiency (EQE) of All of the Samples Measured

	in Figure 3a					in Figure 3c	
sample	fresh	4 days	5 days	6 days	7 days	9 months	12 months
**EQE**	0.029%	0.043%	0.027%	0.019%	0.005%	0.123%	0.037%

The sequence of photoinduced and redox processes responsible for hydrogen generation and iodide oxidation during photocatalysis with 4FPSI is represented by the following reactions:

$$4\mathrm{FPSI}+h\nu \to e^{-}+h^{+}$$

$$2H^{+}+2e^{-}\to H_{2}$$

$$2I^{-}+2h^{+}\to I_{2};\;I_{2}+I^{-}\to I_{3}^{-}$$

$$I_{2}+H_{3}{\mathrm{PO}}_{2}+H_{2}O\to H_{2}{\mathrm{PO}}_{2}+\mathrm{HI}$$

The observed enhancement in activity upon intermittent irradiation, see Figure 2, and after reusing, see Figure 3, led us to examine the role of surface termination on the photocatalytic behavior. In 2DLHP, the photocatalytic activity is extremely sensitive to surface structure and chemistry, as small variations in termination can shift band edges, modulate surface dipoles, and influence charge-carrier localization.^31,32^ To explore these effects, we modeled three distinct slab configurations using the HSE06 hybrid functional. These included a Sn-terminated surface with exposed undercoordinated tin atoms (Sn-term), a surface fully passivated by organic ligands (Org-term), and a mixed termination composed of an organic group coupled with an apical iodine vacancy (Mixed-term), see Figure 4a.

**Figure 4:**
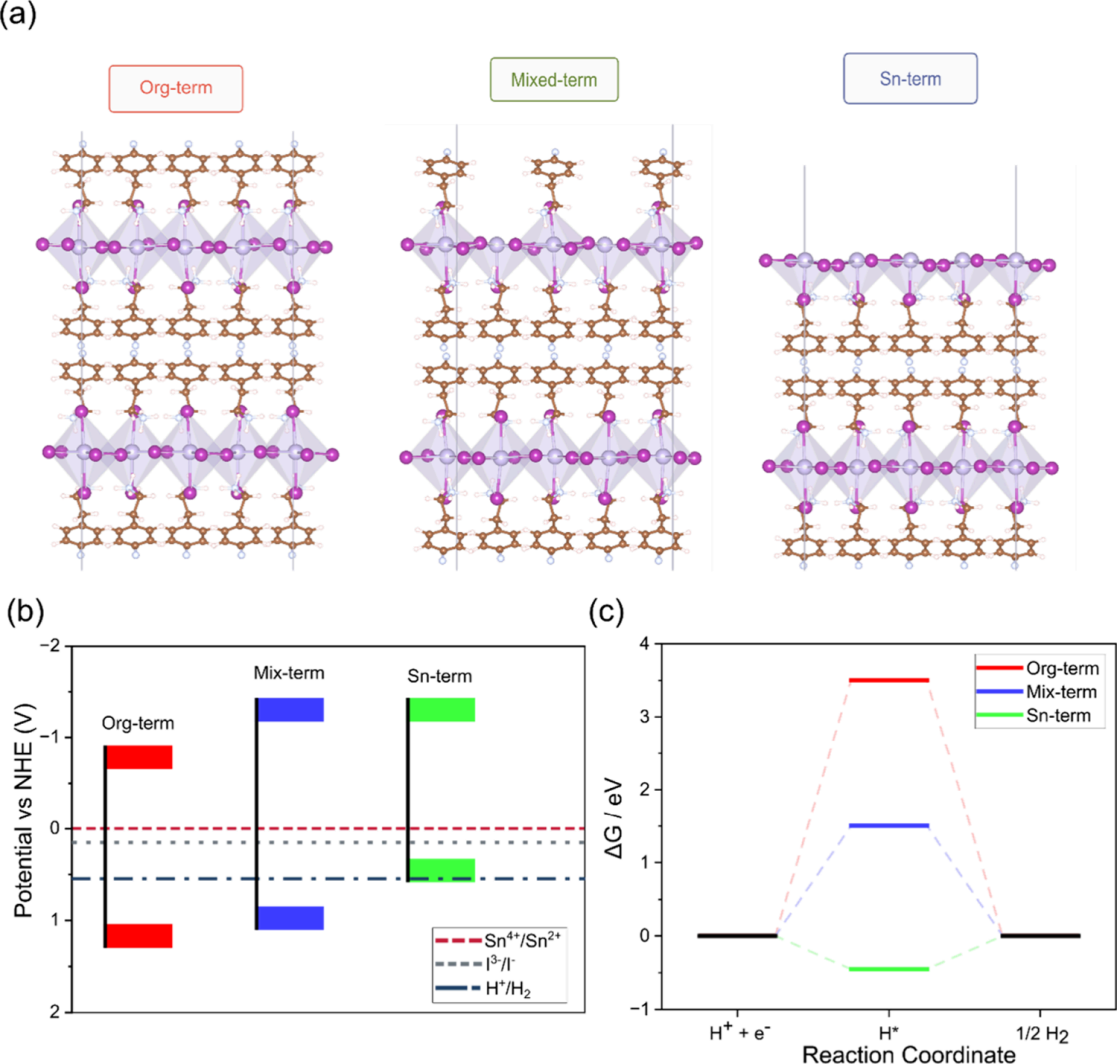
(a) Side-view atomic structures of the three surface terminations, Org-term, Mixed-term, and Sn-term. (b) Absolute VB and CB edge positions vs NHE. (c) Free-energy diagrams for proton adsorption (Δ*G*_H*_) on each termination, indicating the thermodynamic driving force for hydrogen evolution.

Our simulations, see Figure 4b, reveal that all three surfaces possess conduction bands sufficiently negative to drive proton reduction, CBs from −0.91 to −1.43 V vs NHE, while their valence bands are positive enough to oxidize iodide, I^–^ → I_2_ + 2e^–^, E° = +0.54 V vs NHE, for photocatalytic HI splitting. The Sn-term surface shows the most suitable band alignment and exhibits moderate hydrogen binding strength, Δ*G*_H*_ = −0.45 eV, see Figure 4c, which is closer to the optimal range for H_2_ evolution compared to the mixed and organic terminations that show prohibitively weak H* binding, Δ*G*_H*_ = +1.5 eV and +3.5 eV, for mixed and organic terminations, respectively. Chemical-potential–dependent surface formation energy simulated under iodide-rich conditions shows the organic terminated surface is the most stable, γ ≈ −0.95 J/m^2^, followed by mixed, γ ≈ −0.80 J/m^2^, and Sn-terminated, γ ≈ −0.65 J/m^2^, surfaces.^33^

However, the Sn-terminated surface becomes increasingly favorable, suggesting that light exposure or partial HI loss during intermittent irradiation may shift the surface equilibrium toward more catalytically active Sn-rich domains. Notably, the active sites for H_2_ photoproduction are associated with exposed tin atoms, which emerge due to surface distortions during the reaction process, as illustrated in Figure S5. These structural rearrangements elevate Sn atoms above the surface, enhancing their accessibility and reactivity. Despite this enhanced reactivity, the defective surfaces are more susceptible to oxygen/tin interactions, leading to gradual oxidation. This oxidation process compromises the catalytic efficiency by passivating the active sites. This mechanistic understanding endorses the photocatalytic enhancement in H_2_ evolution, with the maximum activity observed after moderate aging and a subsequent decrease associated with Sn–O interactions and active site passivation. Such behavior highlights the critical role of controlled aging in optimizing photocatalytic performance. This mechanistic understanding aligns with the observed trend of initially improved H_2_ evolution performance upon aging and repeated use, followed by eventual decrease.

### Degradation Mechanism

After five photocatalytic tests, the as-prepared samples were degraded. The degradation mechanism of tin perovskites has been described by Lanzetta et al.^25^ Accordingly, 4FPSI is known to degrade into various oxidized products, including 4FPEAI, SnI_4_, SnO_2_, and (4FPEA)_2_SnI_6_, among others. XRD patterns comparing the fresh and degraded samples are listed in Figure 5a. The microstructure of the degraded sample, presented in Figure 5b, revealed hollowed hexagonal features that suggest a structural collapse. This degradation process led to the formation of a white-colored turbid solution, see Figure S6, consistent with the formation of oxidized tin species upon the slow hydration of the Sn perovskite. From a theoretical standpoint, our DFT simulations further reveal how the oxidation of Sn on the surface changes its coordination environment from an octahedral shape to a more planar one. These structural distortions are particularly pronounced when Sn is oxidized by two oxygen atoms, and they are consistent with the increased disorder observed in XRD patterns. Moreover, the oxidized tin centers exhibit a stronger affinity toward adsorbed oxygen species compared to iodine. This results in a cleavage of the interaction with iodine from the bottom layer, further contributing to a loss of lattice coherence, see Figure S5b. Once oxygen atoms are bonded to Sn, they generate local symmetry breaking and lattice destabilization. This atomistic picture supports the idea that oxidation-induced geometric destabilization is a primary driver of the degradation of the material.

**Figure 5:**
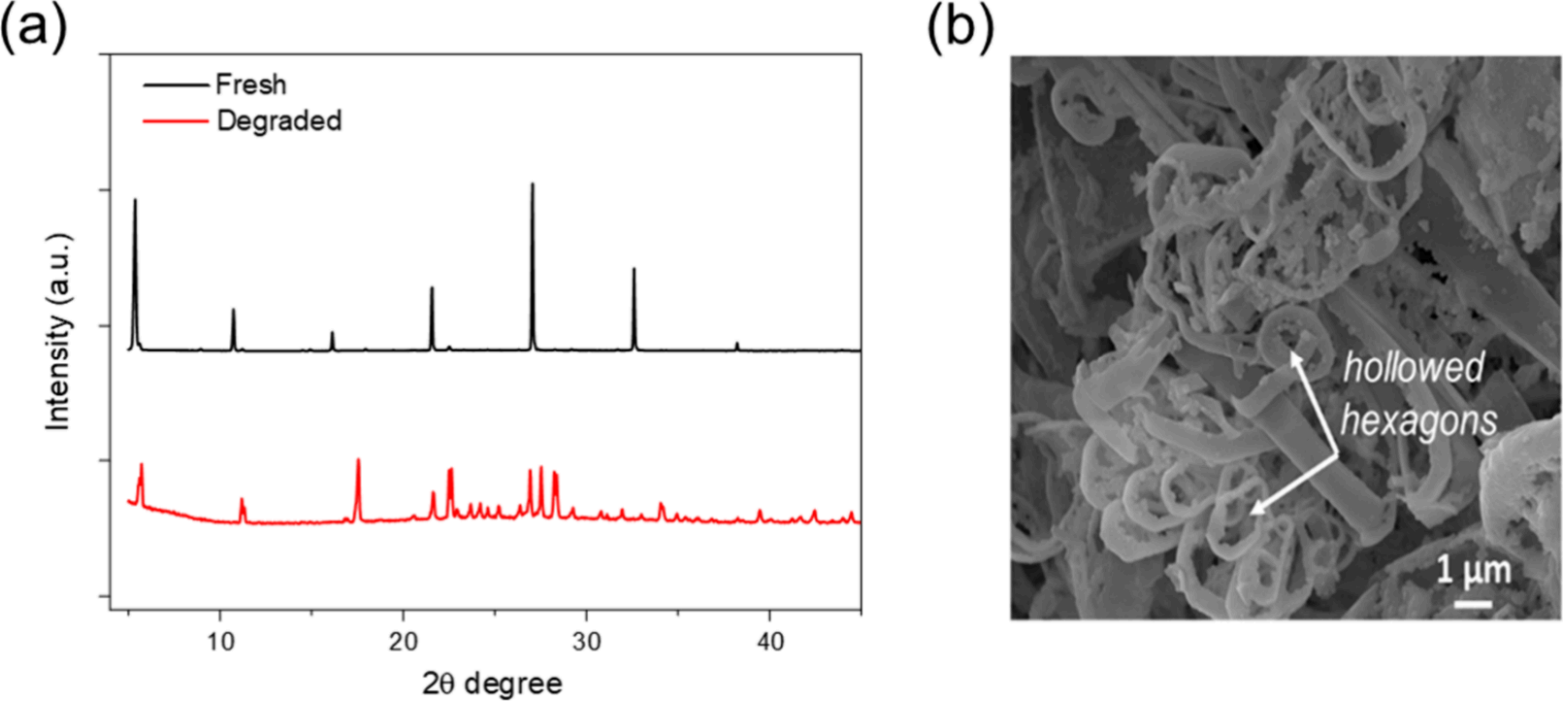
(a) XRD patterns of fresh 4FPSI (black) and its degraded product (red). (b) SEM image of the degraded product.

### Recovery of the Degraded Perovskite

The degraded products (and side products) containing Sn^4+^ can be regenerated by reduction to Sn^2+^ by adding HI+H_3_PO_2_. When the degraded solid is treated with this solution and heated, water evaporates, and Sn^4+^ is reduced to Sn^2+^. Upon cooling, a spontaneous reaction between 4FPEA^+^ and [SnI_4_]^2–^ complexes occurs, leading to the precipitation of red 4FPSI MCs. Although the aim of heating is to redissolve all the degraded products, a residual solid remains undissolved. This residue is likely Sn­(OH)_2_, a hydrolysis product formed from the reaction between SnO_2_ and HI. Since Sn­(OH)_2_ is poorly soluble, HCl was introduced to the system to aid dissolution. The protons (H^+^) from HCl neutralize hydroxide groups (OH^–^), forming water and a soluble tin halide salt. Heating this solution (HI + HCl) to 150 °C produces a clear, transparent solution, indicating complete dissolution. After 15 min of heating to evaporate excess solvent, rapid cooling yielded the formation of red-colored 4FPSI MCs, see Figure S6.

The recrystallization mechanism follows three main steps:

1. Dissolution and reduction—Degraded products are treated with HI + H_3_PO_2_ (with or without HCl) and heated to 150 °C to reduce Sn^4+^ to Sn^2+^ and dissolve all precursors.
2. Supersaturation and nucleation—Continued heating evaporates solvents, pushing the system to supersaturation. Upon cooling, the nucleation begins.
3. Crystal growth—Rapid cooling drives fast crystal growth, yielding microcrystalline 4FPSI.

To probe the self-healing potential under reducing conditions, we simulated a defective 4FPSI system in the presence of an excess of hydroiodic acid (HI). The HI-rich environment reduces adsorbed oxygen species to H_2_O, enabling the progressive reincorporation of iodine atoms. Our simulations, see Figure 6, show that while the protons are prone to reduce the system, iodine counterparts can repopulate both top surface sites and interstitial sites within the perovskite lattice, facilitating defect passivation.

**Figure 6:**
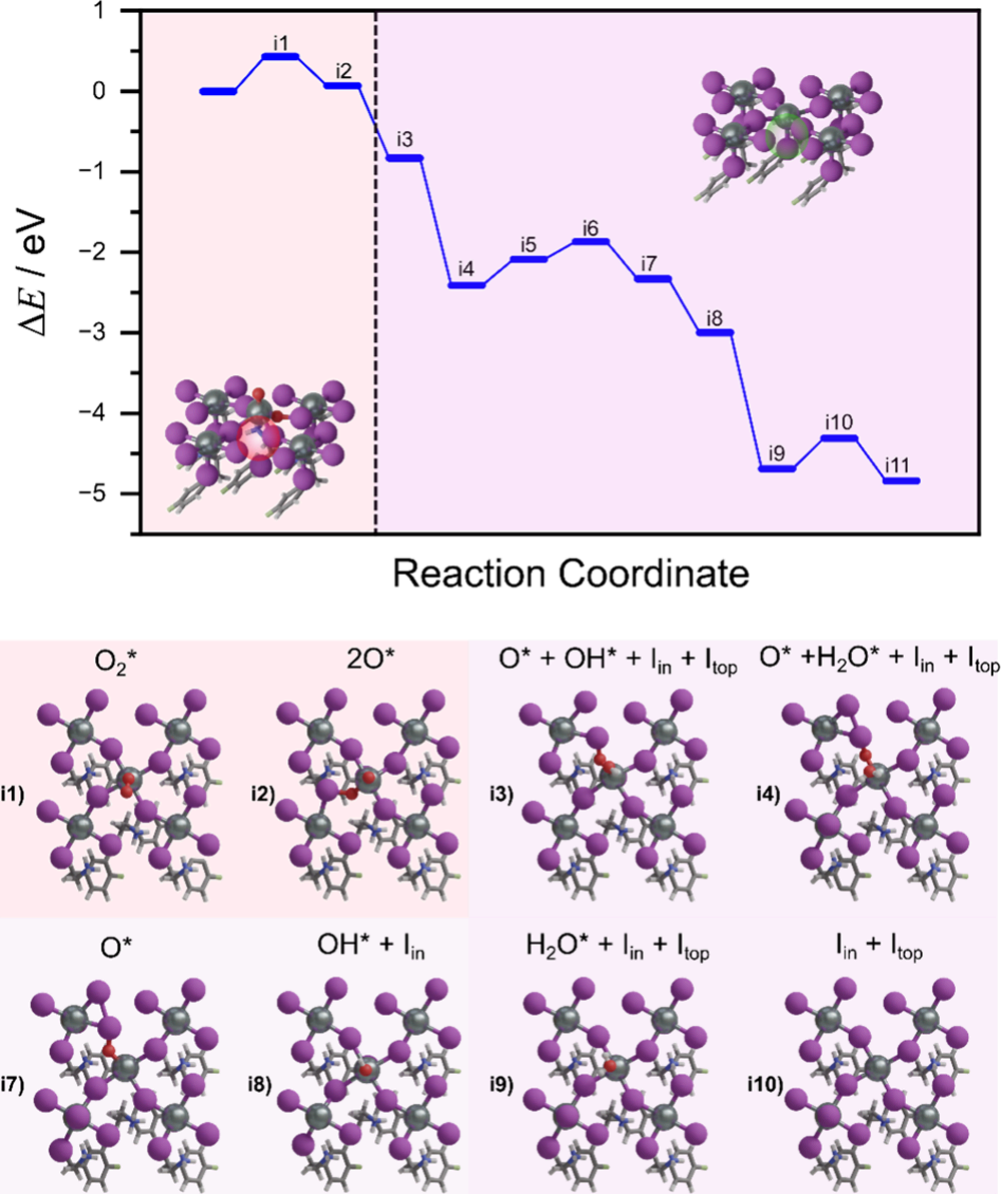
(top) Reaction pathway of the oxygen-induced defects and intermediate states for self-healing in tin halide perovskite: from O_2_ adsorption to halide recovery. Orange: oxygen-rich environment. Light-purple: HI-rich environment. Green and red circles indicate the presence or absence (respectively) of the Sn–I bond, showing if it maintains the octahedral shape. (bottom) Structural models of the intermediates are defined in the top reaction coordinate. Color code: Sn: metallic, I: purple, oxygen: red, carbon: gray, nitrogen: blue, hydrogen: white.

The healing mechanism proceeds through several stable intermediates, culminating in full recovery of the iodine-deficient lattice. The reduction of the surface goes by the hydrogenation of the oxygens, while the iodine counterparts are going to the top or interstitial sites. As it reacts, one water molecule is released to the medium and the surface suffers a reconstruction releasing I_2_, i4 of Figure 6. The reduction of the second oxygen occurs similarly by healing the catalyst. Once structural healing is achieved, a final rearrangement step is required to reposition Sn atoms back into their original octahedral environment, completing the structural recovery process. This reaction profile demonstrates that chemical reduction via HI not only passivates surface oxygen species but also restores structural coherence by guiding the lattice back to its low-energy configuration. Additionally, the reconstruction of the surface along the pathway suggests that there are two competing tendencies that influence the reactivity. While the reduction of the surface restores the photocatalytic activity, full reconstruction makes the catalyst less reactive when compared to the fresh photocatalyst.

XRD analysis confirms that the recrystallized product is structurally identical to the fresh 4FPSI, suggesting the recovery of the material, see Figure 7a. Moreover, the enhanced PL emission of the recrystallized product further confirms the recovery of the product, see Figure 7b and Figure S7. SEM images of the recrystallized MCs using HI alone, see Figure 7c,d, and HI + HCl, see Figure 7e,f, show well-defined morphologies. To validate the functional recovery, the recrystallized material (from HI only) was tested for photocatalytic HI splitting, and the corresponding H_2_ production performance is shown in Figure S7. Notably, the average H_2_ production is lower compared to fresh samples, likely due to the less reactive reconstructed surface.

**Figure 7:**
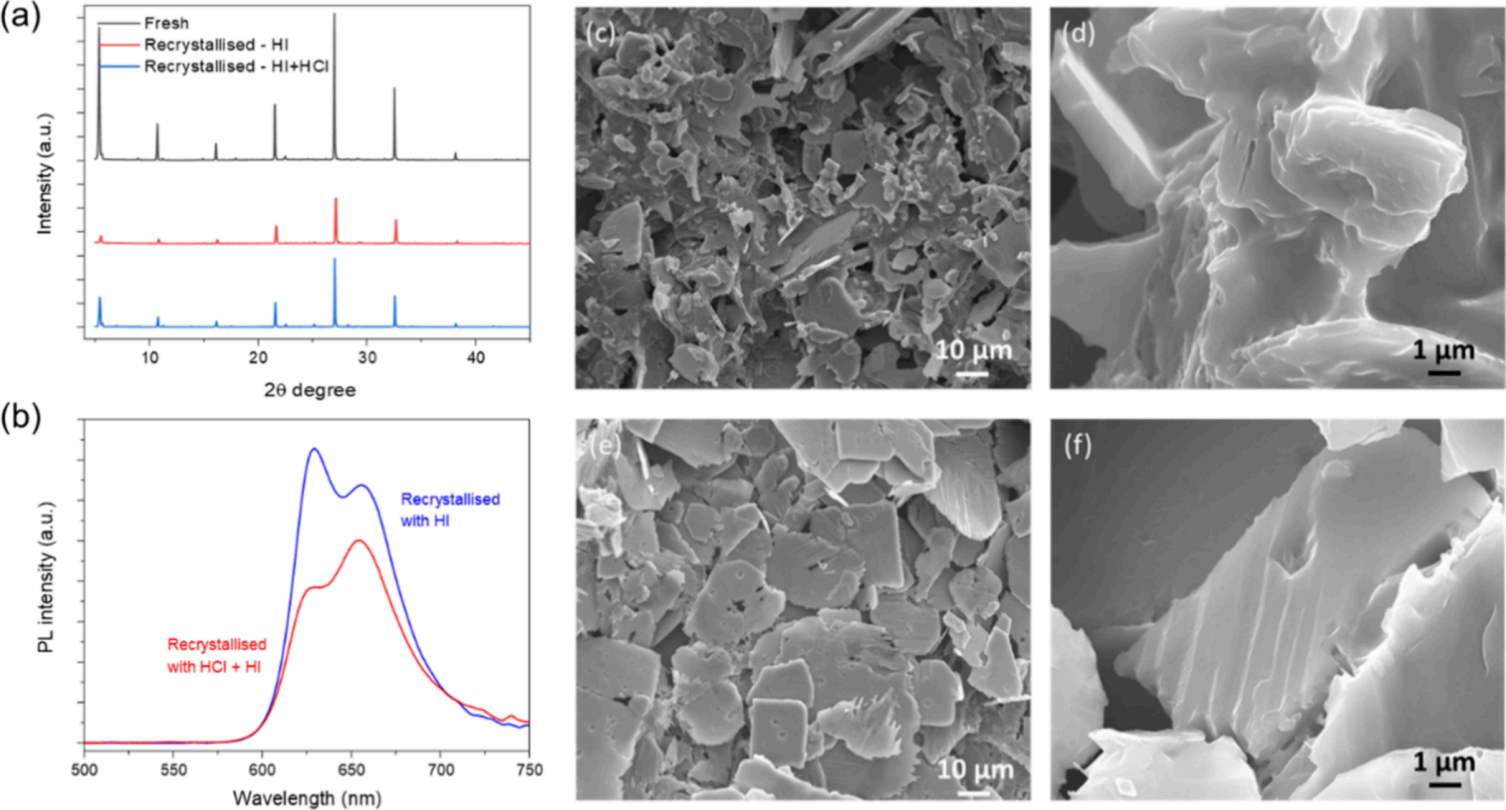
(a) XRD patterns of fresh and recrystallized samples. (b) PL spectra of the recrystallized samples. SEM images of the recrystallized samples using (c, d) HI and (e, f) HI+HCl.

## Conclusions

In this work, 2D 4FSPI perovskite MCs have been synthesized using a HI/H_2_O mixture, yielding materials that remained stable for over 1 year when dispersed in the same solution. Photocatalytic H_2_ production was demonstrated using HI as a sacrificial agent, without the need for any cocatalysts. Intermittent light irradiation significantly enhanced the H_2_ production compared to continuous illumination. Notably, triiodide (typically formed via hole-mediated oxidation of iodide) was not detected probably due to its rapid reduction by H_3_PO_2_ present in the solution. Aged samples showed enhanced photocatalytic performance, relative to the fresh sample, due to the surface reconstruction during dark periods, as supported by DFT. After multiple cycles, the material started to degrade, due to the exposed Sn atoms, making the material more susceptible to oxidative degradation. However, the degraded material could be fully recrystallized and reused for H_2_ production. Moreover, our present study underscores the need for future studies on the degradation mechanism, examining how illumination and environmental conditions influence the stability of tin halide perovskite photocatalysts. In summary, this study demonstrates the development of a chemically robust, aqueous-stable 2D tin-iodide perovskite capable of efficient, catalyst-free HI splitting for H_2_ generation, with an excellent reusability and a viable recovery strategy postdegradation.

## Supplementary Material



## Data Availability

A data set collection of computational results is available in an ioChem-BD repository (https://doi.org/10.19061/iochem-bd-1-385).

## Notes

UV–vis absorbance spectra, standard errors in photocatalytic hydrogen production experiments, reported performance of photocatalytic hydrogen production in lead-free halide perovskites, and energy contributions for H adsorption on 4FPSI (PDF)
