# Supplementary material for: Efficient and Stable Hydrogen Evolution from HI Splitting Using a Robust 2D Tin-Iodide Perovskite
Source: J Phys Chem C Nanomater Interfaces. 2026 Feb 2;130(6):2119–28. doi: 10.1021/acs.jpcc.5c07925 (PMC12908155; doi:10.1021/acs.jpcc.5c07925)
Supplement: Supplementary file 1 [file jp5c07925_si_001.pdf]

Supporting Information

Efficient and Stable Hydrogen Evolution from HI Splitting Using a Robust 2D Tin-Iodide Perovskite

Samiksha Mukesh Jain,[1] Samrat Das Adhikari,[1,2*] Camilo A. Mesa,[1,3] Hind Benzidi,[4] José Manuel González-Acosta,[4,5] Andrés F. Gualdrón-Reyes,[1,6] Núria López,[4*] Sixto Giménez,[1*] Iván Mora-Seró[1*]

[1] Institute of Advanced Materials, Universitat Jaume I, Avinguda de Vicent Sos Baynat, s/n, 12006 Castelló de la Plana, Spain

[2] Institute of Physical Chemistry, Polish Academy of Sciences, Warsaw 01-224, Poland

[3] Catalan Institute of Nanoscience and Nanotechnology (ICN2), CSIC and BIST, Campus UAB, Bellaterra, 08193 Barcelona, Spain

[4] Institute of Chemical Research of Catalonia (ICIQ-CERCA), The Barcelona Institute of Science and Technology (BIST), Av. Països Catalans 16, 43007 Tarragona, Spain

[5] Department of Physical and Inorganic Chemistry, University Rovira I Virgili, Marcel·lí Domingo s/n, 43007 Tarragona, Spain.

[6] Universidad Austral de Chile, Valdivia, Chile

Calculation of External quantum yield (EQE)

EQE was calculated according to the following formula[1] :

$$Solar\ HI\ splitting\ (\%) = \frac{evolved\ H_2\ (mol)\ x\ 6.02 * 10^{23}\ x\ 2\ x\ 0.33\ x\ 1.6 * 10^{-19}}{P_{sol}(W\ cm^{-2})\ x\ Area\ (cm^{-2})\ x\ time\ (s)}\ x\ 100$$

Where,

$P_{sol} = 100\ mW\ cm^{-2}$

Area exposed = 4 cm^2

Modelling section

Computational Methodology

Density functional theory (DFT) calculations were performed to investigate surface defects in the 2D tin-based perovskite 4FPSI. All calculations were carried out using the Vienna Ab initio Simulation Package (VASP)[2], within the projector augmented-wave (PAW) framework[3] and using the Perdew–Burke–Ernzerhof (PBE) functional within the generalized gradient approximation (GGA) [4]. To account for relativistic effects, particularly relevant for Sn atoms, spin–orbit coupling (SOC) was included. Van der Waals interactions were incorporated using the DFT-D3 dispersion correction with zero damping (IVDW = 12)[5], appropriate for layered and hybrid materials.

A plane-wave cutoff energy of 500 eV and Gaussian smearing with a width of 0.05 eV were used. The electronic convergence criterion was set to 10^{-8} eV, while ionic relaxations were carried out using the conjugate gradient algorithm until the maximum force on each atom was less than 0.01 eV/Å. Only atomic positions were relaxed to preserve the slab geometry. For all surface models, sufficient vacuum spacing (>15 Å) was added to eliminate spurious interactions between periodic images. Dipole corrections were considered where necessary to account for asymmetry in the slab terminations. The Monkhorst–Pack k-point mesh was adjusted based on slab size and symmetry; for large surface supercells, a Γ-point or reduced grid was used to ensure convergence of total energies and defect states. Charge densities and wavefunctions were saved for further analysis of surface states and absorbers.

In this study, we modeled intrinsic point defects in $4F\text{-}PEA_2SnI_4$ using a 2×2×2 supercell for point defects and 2x2x1 for reactivity. Point defects including vacancies (V_{Sn} , V_I) and interstitials (Sn_i, I_i). Vacancies refer to missing atoms at regular lattice sites V_{Sn} and V_I denote tin and iodine vacancies, respectively, typically acting as acceptor or donor defects. Interstitials represent atoms occupying non-lattice positions: Sn_i^{in} and I_i^{in} are atoms inserted into bulk

interstitial sites, while Sn_i^{out} and I_i^{out} are displaced toward the outer regions of the lattice, often influencing surface energetics or forming metastable configurations. These defects are critical to understanding charge carrier dynamics, recombination, and the overall stability of 2D perovskites.

The defect formation energy for a point defect X in charge state q was calculated as:

$$E_f(X_q) = E_{tot}^{defected} - E_{tot}^{perfect} + \sum n_i \, \mu_i + q(E^{VBM} + E^{Fermi} + \Delta V)$$

where:

- $E_{tot}^{defected}$ and $E_{tot}^{perfect}$ the total energies of the defected and pristine supercells, respectively. n_i represents the number of atoms of species i added or removed.
- μ_i is the chemical potential of species i. q is the charge state of the defect.
- E_{VBM} is the valence band maximum.
- E_{Fermi} is the Fermi level referenced to the VBM.
- ΔV accounts for potential alignment corrections.

The chemical potentials were constrained by the thermodynamic stability of 4FPSI with respect to competing phases such as SnI_2 and SnI_4. The allowed range of chemical potential ensures the absence of secondary phases in the stability window.

Band alignment was performed using the vacuum level alignment method. Electrostatic potentials and charge densities were extracted to determine the absolute positions of the valence band maximum (VBM) and conduction band minimum (CBM) relative to the vacuum level.

Convergence tests were carried out with respect to plane-wave cutoff energy, k-point sampling, and supercell size, ensuring reliable values for defect formation energies and band edge positions. This methodology provides essential insights into the defect energetics and band alignment in 4F-PEA_2SnI_4, contributing to its optimization for optoelectronic and photovoltaic applications.

Surface Structure and Termination Models

To investigate the effect of surface chemistry on band-edge alignment in our 2D Sn-perovskite, we constructed three slab models that capture realistic terminations: (i) undercoordinated Sn

atoms exposed at a Sn-terminated surface, (ii) full coverage by two organic cations (Org-terminated), and (iii) a mixed termination with one organic cation and one missing apical iodine (Mixed-terminated) (Figure 4a in the main text). Each slab was relaxed until forces fell below 0.01 eV $Å^{-1}$, then a single-point HSE06 simulation[6] was performed to obtain the Kohn–Sham eigenvalues and plane-averaged electrostatic potential. The vacuum level was identified as the flat plateau in electrostatic potential and referenced against the bulk-like interior[7]. Absolute band-edge energies were computed by subtracting this vacuum level from the VBM and CBM eigenvalues, yielding E^{abs} relative to vacuum. Finally, these absolute energies were converted to potential versus the normal hydrogen electrode via

$$E_{vsNHE} = -\,(E^{abs} + 4.44eV)$$

where 4.44 eV is the vacuum–NHE offset at pH 0.[8] The resulting VBM and CBM potentials were compared to standard redox couples (H^+/H_2 at 0 V, I_3^-/I^- at +0.54 V, O_2/H_2O at +1.23 V vs NHE) to assess photocatalytic viability.

Hydrogen Evolution Activity and Gibbs Free Energy

The HER activity of different surface terminations in 4FPSI was evaluated by calculating the Gibbs free energy of hydrogen adsorption (ΔG_{H^*}), a widely accepted descriptor for catalytic performance[9]. The free energy change associated with the adsorption of a hydrogen atom on the surface was calculated as:

$$\Delta G_{H^*} = \Delta E_{H^*} + \Delta ZPE - T\Delta S$$

whereΔE_{H^*} is the adsorption energy of hydrogen, defined as the total energy difference between the H-adsorbed surface and the clean surface plus half of an H_2 molecule. The zero-point energy correction (ΔZPE) and the entropy contribution (TΔS) were computed using vibrational analysis through the Phonopy code[10](See table S2), based on the finite displacement method

and forces obtained from VASP. Only the adsorbed H atom was displaced during vibrational analysis, with the surface slab kept fixed. The temperature (T) was set to 298.15 K. Surfaces with ΔG_{H^*} values close to zero are considered optimal for HER, as they balance hydrogen adsorption and desorption. All calculated frequencies were real, confirming that each H* configuration corresponds to a stable local minimum. Only H* vibrational modes were used for zero-point energy and entropy corrections. This methodology allows for a comparative assessment of HER catalytic potential across various terminations, correlating surface chemistry, defect states, and photocatalytic suitability.

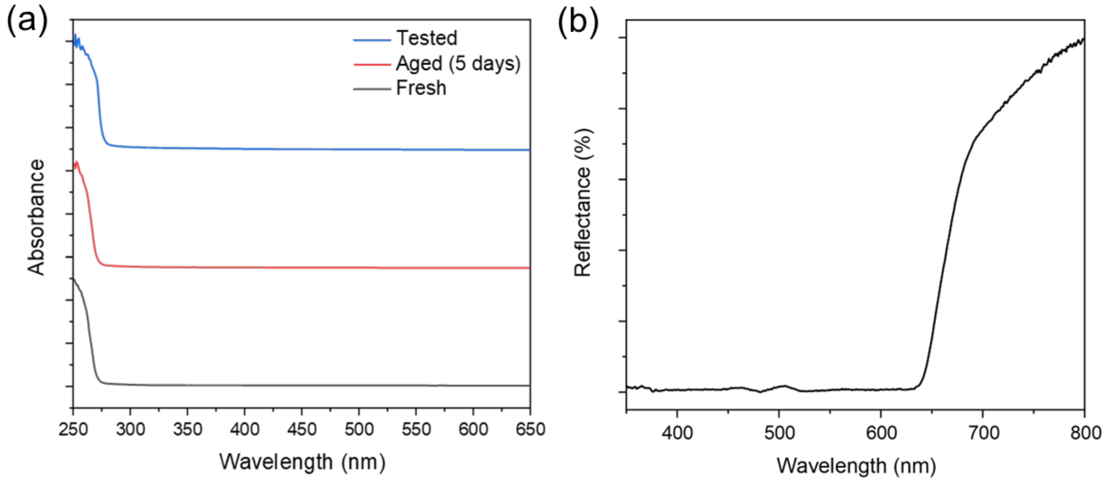

Figure S1. (a) Absorbance spectra of the supernatant solution. As-prepared sample (black plot), aged sample for 5 days (red plot), and tested catalyst (blue plot). No triiodide characteristic absorption peaks at ~288 nm and ~350 nm were detected as oxidation product and (b) UV-Vis diffused reflectance spectrum of the as-prepared sample.

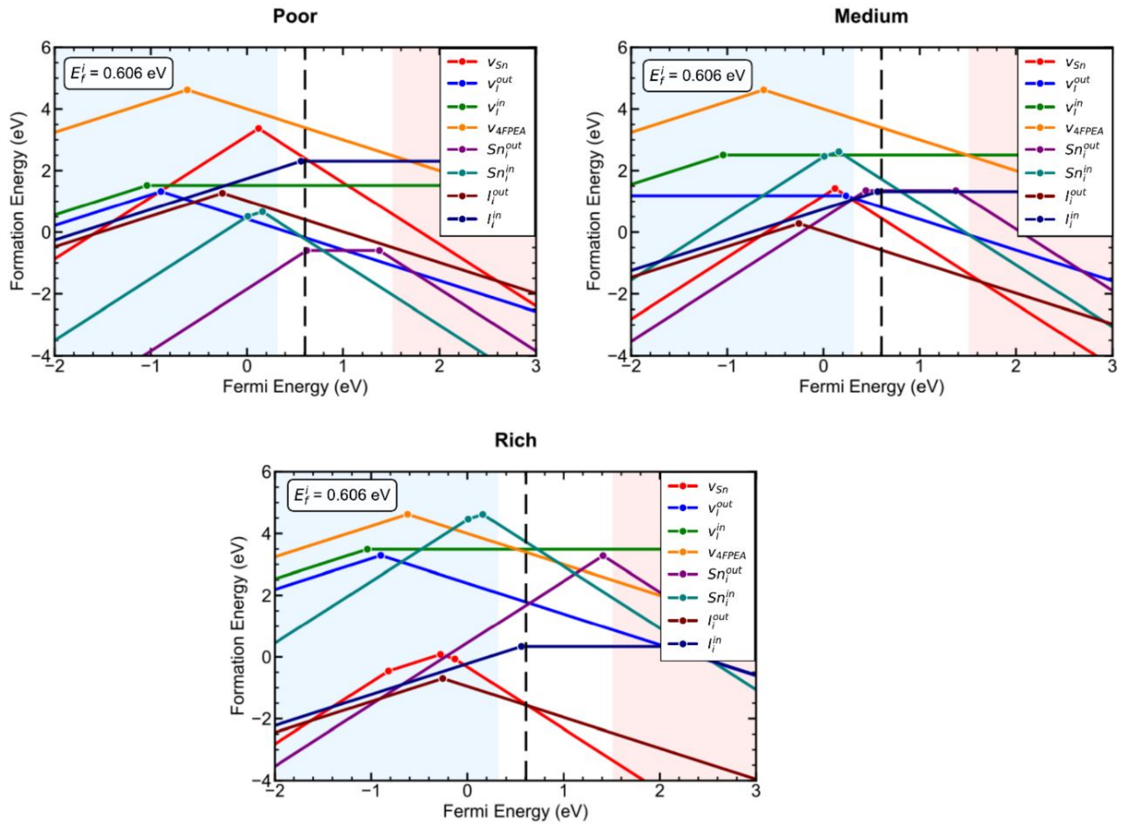

Figure S2. Fermi Level Dependent Defect Formation Energies in Tin Halide Perovskite under Poor, Medium, and Rich Growth Conditions.

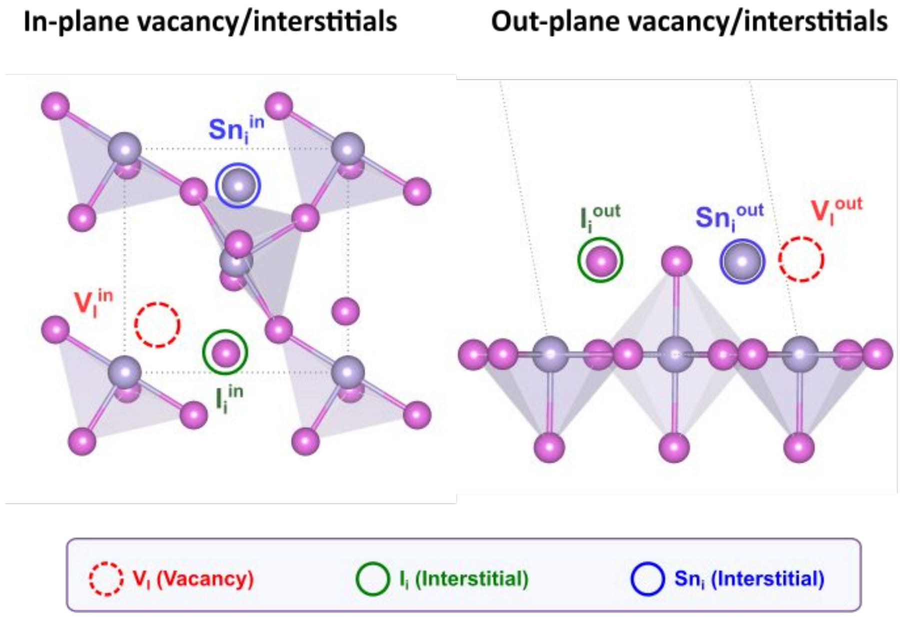

Figure S3. Schematic illustration of in-plane and out-of-plane point defects in the SnI_6 octahedral framework of 4FPSI.

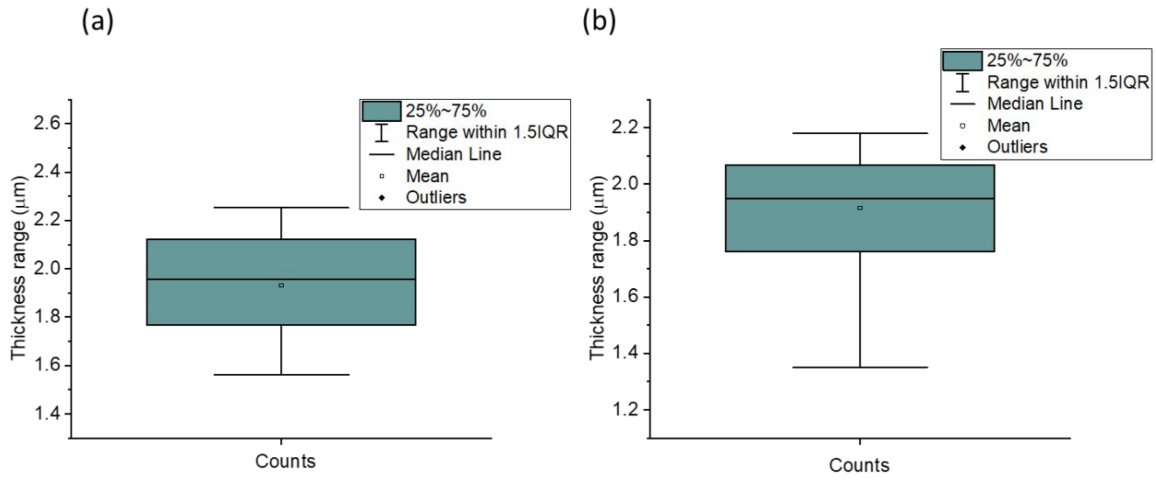

Figure S4. 4FPSI microplatelet thickness of (a) freshly prepared, and (b) tested sample, estimated from SEM measurements.

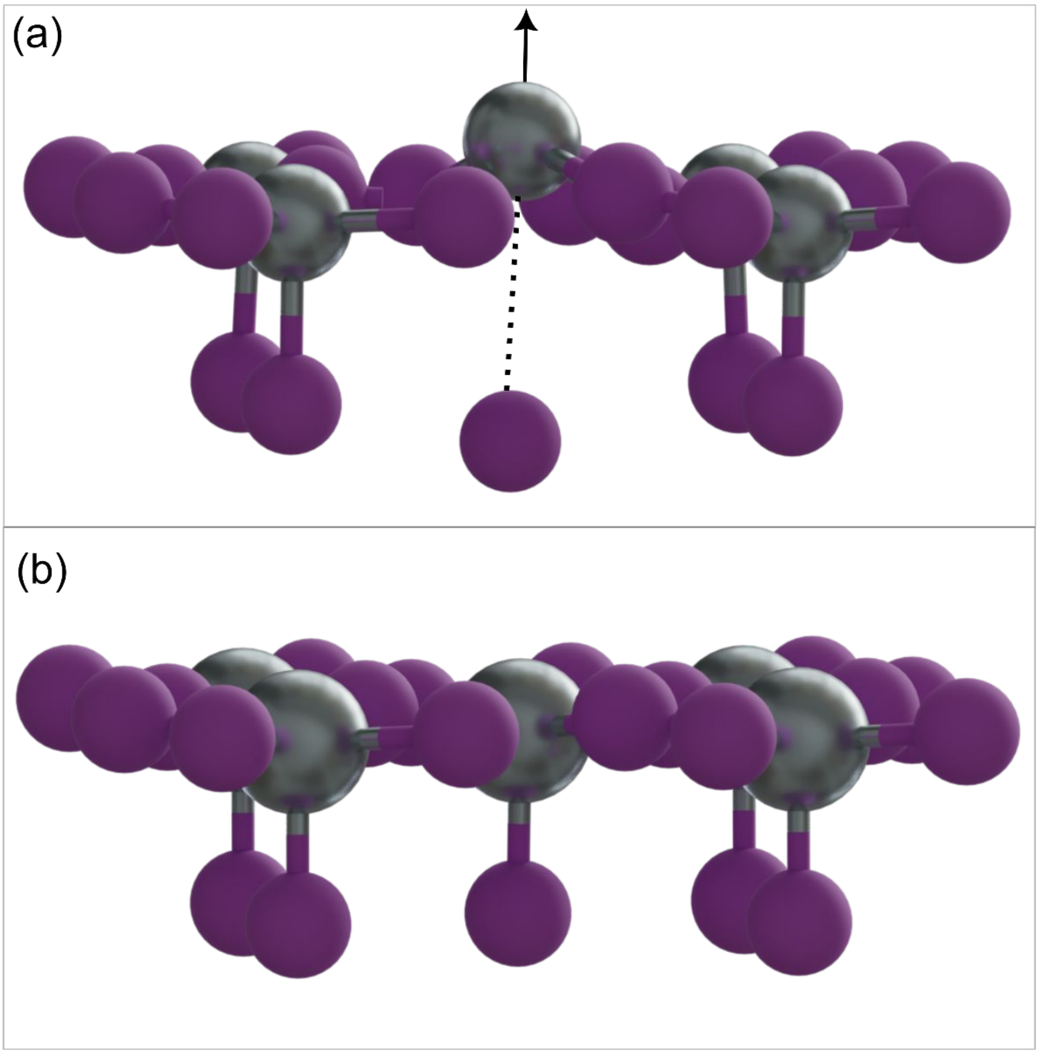

Figure S5. (a) Model of the Sn-terminated perovskite surface before healing, showing the distortion on a Sn atom. The oxidation of Sn distorts its octahedral coordination, leading to the cleavage of the Sn–I bond and displacement of the Sn atom out of the surface plane. (b) Restored surface model after healing.

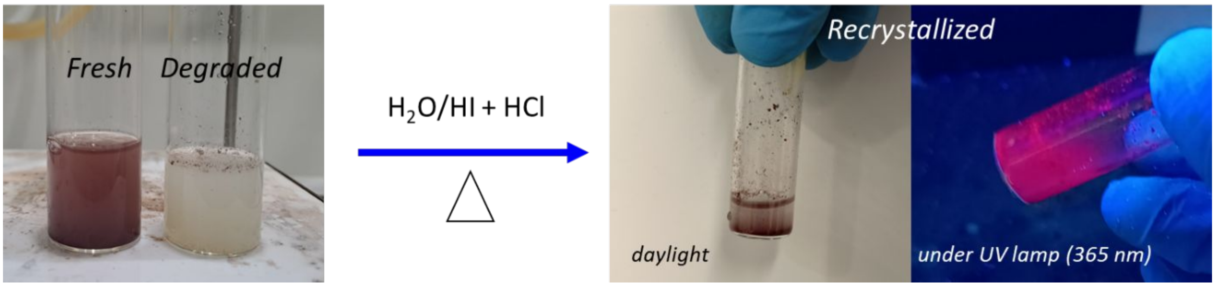

Figure S6 Digital photograph of the stable 4FPSI suspension in HI/H_2O, and degraded dispersion after 5 times of photocatalytic tests, and the recrystallized product from the degrade sample under daylight and UV lamp.

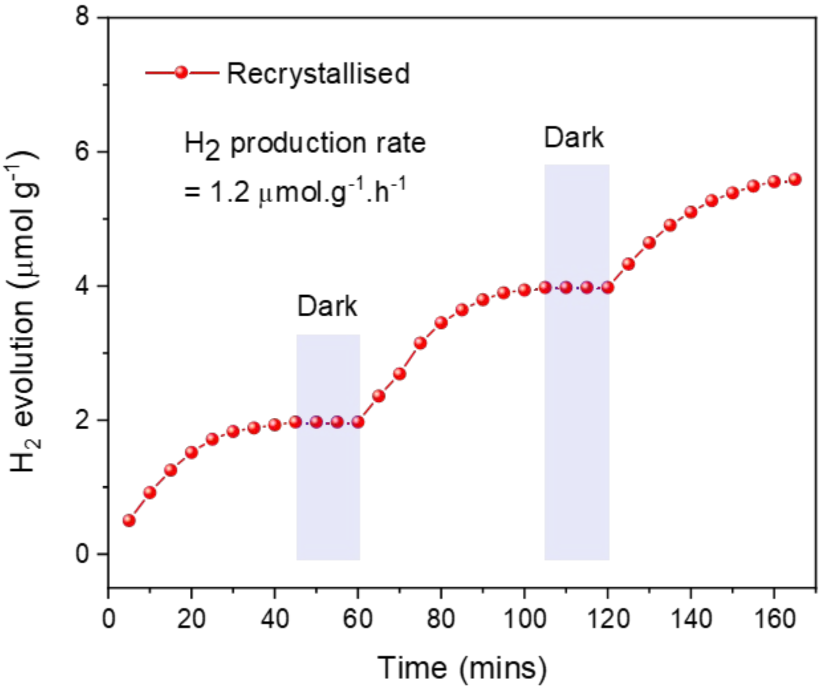

Figure S7. Photocatalytic H_2 production of recrystallized 4FPSI microcrystals using HI.

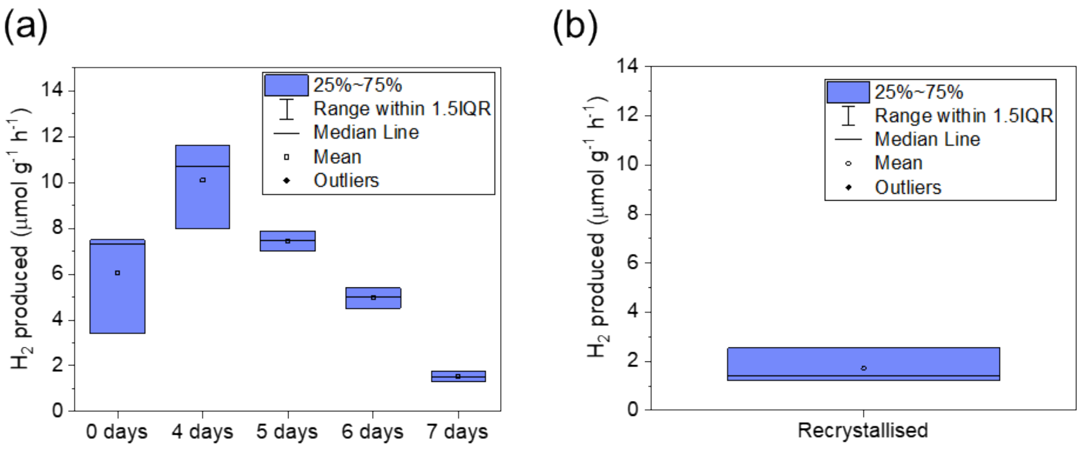

Figure S8. Photocatalytic H_2 production in (a) sample studying ageing effect, and (b) recrystallized sample, with error bars representing standard errors from 3 experiments

Table S1 – Comparison of photocatalytic hydrogen evolution performance between this work and previously reported lead-free halide perovskites.

No.	Material	Additive /Co-catalyst	Amount of hydrogen generated	Stability	Reference
**1**	4FPSI	-	Fresh - 7.3µmol g^{-1} h^{-1}	12 months	**This work**
**2**	Cs_2SnBr_6	Pt	Pristine Cs_2SnBr_6 – 0.8 mmol g^{-1} h^{-1}<br><br>$Cs_2Pt_{0.25}Sn_{0.75}Br_6$ – 11.49 mmol g^{-1} h^{-1}<br><br>Cs_2SnBr_6-Pt – <2 mmol g^{-1} h^{-1}	Pristine Cs_2SnBr_6 – 10 days<br><br>$Cs_2Pt_{0.25}Sn_{0.75}Br_6$ – 30 days	11
**4**	$Cs_2Pt_xSn_{1-x}Cl_6$	Pt doped	$Cs_2Pt_{0.05}Sn_{0.95}Cl_6$ – 16 µmol g^{-1} h^{-1}<br><br>$Cs_2Pt_{0.50}Sn_{0.50}Cl_6$ – 3.99 µmol g^{-1} h^{-1}<br><br>$Cs_2Pt_{0.75}Sn_{0.25}Cl_6$ – 0 µmol g^{-1} h^{-1}	25 days	12
**5**	Cs_2SnI_6	$Pt-I_3$	Pristine Cs_2SnI_6 – 25 µmol g^{-1} h^{-1}<br><br>$Pt-I_{\#}$/ Cs_2SnI – 430 µmol g^{-1} h^{-1}<br><br>Photo-deposited Pt over Cs_2SnI_6 – 101 µmol g^{-1} h^{-1}	4 cycles, each cycle measured for 6h	13

**7**	$DMASnBr_3$	Graphitic carbon nitride	33% $DMASnBr_3$@g-C_3N_4 (pure water, no Pt) – 7 µmol g^{-1} h^{-1}<br><br>33% $DMASnBr_3$@g-C_3N_4 (pure water, 3% Pt) - 14 µmol g^{-1} h^{-1}<br><br>33% $DMASnBr_3$@g-C_3N_4 (10% TEOA, 3% Pt) – 1730 µmol g^{-1} h^{-1}	32h	[14]
**8**	PEA_2SnBr_3	Graphitic carbon nitride	Pristine perovskite – 4 µmol g^{-1} h^{-1}<br><br>Composite – 1600 µmol g^{-1} h^{-1}	4h	[15]
**9**	DMA_xSnBr_3	Pt	DMA_xSnBr_3 – 6 µmol g^{-1} h^{-1}<br><br>With TEOA and 1%wt Pt - 11 µmol g^{-1} h^{-1}	6 days	[16]
**10**	$DMASnI_3$	-	$DMASnI_3$ – 0.64µmol g^{-1} h^{-1}	16h	[17]
**11**	$DMASnBr_3$	g-C_3N_4 and Pt	925 µmoles g^{-1} h^{-1}		[18]
**12**	$Cs_2AgBiBr_6$	Nitrogen doped Carbon	$Cs_2AgBiBr_6$ – 20 µmol g^{-1} h^{-1}<br><br>$Cs_2AgBiBr_6$/N-C – 380 µmol g^{-1} h^{-1}	6 cycles, each cycle measured for 4h	[19]
**13**	$Cs_3Bi_{2x}Sb_{2-2x}I_9$	Pt	$Cs_3Bi_{2x}Sb_{2-2x}I_9$/Pt – 92.6 µmol g^{-1} h^{-1}	50h	[20]
**14**	$Cs_2AgBiBr_6$	RGO and Pt	$Cs_2AgBiBr_6$ – 6.1 µmol g^{-1}<br><br>$Cs_2AgBiBr_6$/Pt – 9.5 µmol g^{-1}<br><br>$Cs_2AgBiBr_6$/RGO – 489 µmol g^{-1}	12 cycles, each cycle measured for 10h	[21]
**15**	$MA_3Bi_2I_9$	Pt	$MA_3Bi_2I_9$ – 12.19 µmol g^{-1} h^{-1}<br><br>$MA_3Bi_2I_9$/Pt – 169.21 µmol g^{-1} h^{-1}	7 cycles, each cycle measured for 10h	[22]

Table S2. Vibrational energy and entropy contributions for H* adsorption on 4FPSI terminations (finite-displacement phonons, 298 K).

	E_ZPE (eV)	U(T) (eV)	H(T) (eV)	G(T) (eV)	Entropy (eV/K)
**Org-term**	0.023535	0.032507	0.032507	0.019053	0.000045
**Sn-term**	0.145537	0.167362	0.167362	0.132824	0.000116
**Mix-term**	0.022870	0.056199	0.056199	-0.019818	0.00025

References

(1) Park, S.; Chang, W. J.; Lee, C. W.; Park, S.; Ahn, H. Y.; Nam, K. T. Photocatalytic Hydrogen Generation from Hydriodic Acid Using Methylammonium Lead Iodide in Dynamic Equilibrium with Aqueous Solution. *Nat Energy* 2017, *2* (1). https://doi.org/10.1038/nenergy.2016.185.

(2) Kresse, G.; Furthmü, J. *Efficient Iterative Schemes for Ab Initio Total-Energy Calculations Using a Plane-Wave Basis Set*; Physical Reviews B, 1996; Vol. 54. https://doi.org/10.1103/PhysRevB.54.11169.

(3) Blochl, P. E. Projector Augmented-+rave Method. *PHYSICAL REVIEW B VOLUME* 1994, *50*, 17953–17979. https://doi.org/10.1103/PhysRevB.50.17953.

(4) Perdew, J. P.; Burke, K.; Ernzerhof, M. *Generalized Gradient Approximation Made Simple*; 1996.

(5) Grimme, S.; Antony, J.; Ehrlich, S.; Krieg, H. A Consistent and Accurate Ab Initio Parametrization of Density Functional Dispersion Correction (DFT-D) for the 94 Elements H-Pu. *Journal of Chemical Physics* 2010, *132* (15). https://doi.org/10.1063/1.3382344.

(6) Heyd, J.; Scuseria, G. E.; Ernzerhof, M. Hybrid Functionals Based on a Screened Coulomb Potential. *Journal of Chemical Physics* 2003, *118* (18), 8207–8215. https://doi.org/10.1063/1.1564060.

(7) Goniakowski, J.; Finocchi, F.; Noguera, C. Polarity of Oxide Surfaces and Nanostructures. *Reports on Progress in Physics* 2008, *71* (1). https://doi.org/10.1088/0034-4885/71/1/016501.

(8) Sergio Trasatti. The Absolute Electrode Potential: An Explanatory Note. *J Electroanal Chem Interfacial Electrochem* 1986, *209* (2), 417–428. https://doi.org/10.1016/0022-0728(86)80570-8.

(9) Nørskov, J. K.; Bligaard, T.; Logadottir, A.; Kitchin, J. R.; Chen, J. G.; Pandelov, S.; Stimming, U. Trends in the Exchange Current for Hydrogen Evolution. *J Electrochem Soc* 2005, *152* (3), J23. https://doi.org/10.1149/1.1856988.

(10) Togo, A.; Chaput, L.; Tadano, T.; Tanaka, I. Implementation Strategies in Phonopy and Phono3py. *Journal of Physics Condensed Matter*. Institute of Physics September 4, 2023. https://doi.org/10.1088/1361-648X/acd831.

(11) Wang, J.; Zhang, M.; Chen, Z.; Li, L.; Jiang, G.; Li, Z. Enabling Enhanced Photocatalytic Hydrogen Evolution in Water by Doping Cs2SnBr6 Perovskite with Pt. *ACS Energy Lett* 2024, *9* (2), 653–661. https://doi.org/10.1021/acsenergylett.4c00144.

(12) Yin, H.; Chen, J.; Guan, P.; Zheng, D.; Kong, Q.; Yang, S.; Zhou, P.; Yang, B.; Pullerits, T.; Han, K. Controlling Photoluminescence and Photocatalysis Activities in Lead-Free Cs2PtxSn1−xCl6 Perovskites via Ion Substitution. *Angewandte Chemie - International Edition* 2021, *60* (42), 22693–22699. https://doi.org/10.1002/anie.202108133.

(13) Zhou, P.; Chen, H.; Chao, Y.; Zhang, Q.; Zhang, W.; Lv, F.; Gu, L.; Zhao, Q.; Wang, N.; Wang, J.; Guo, S. Single-Atom Pt-I3 Sites on All-Inorganic Cs2SnI6 Perovskite for Efficient Photocatalytic Hydrogen Production. *Nat Commun* 2021, *12* (1). https://doi.org/10.1038/s41467-021-24702-8.

(14) Romani, L.; Speltini, A.; Ambrosio, F.; Mosconi, E.; Profumo, A.; Marelli, M.; Margadonna, S.; Milella, A.; Fracassi, F.; Listorti, A.; De Angelis, F.; Malavasi, L. Water-Stable DMASnBr3 Lead-Free Perovskite for Effective Solar-Driven Photocatalysis. *Angewandte Chemie - International Edition* 2021, *60* (7), 3611–3618. https://doi.org/10.1002/anie.202007584.

(15) Romani, L.; Bala, A.; Kumar, V.; Speltini, A.; Milella, A.; Fracassi, F.; Listorti, A.; Profumo, A.; Malavasi, L. PEA2SnBr4: A Water-Stable Lead-Free Two-Dimensional Perovskite and Demonstration of Its Use as a Co-Catalyst in Hydrogen Photogeneration and Organic-Dye Degradation. *J Mater Chem C Mater* 2020, *8* (27), 9189–9194. https://doi.org/10.1039/d0tc02525a.

(16) Pisanu, A.; Speltini, A.; Quadrelli, P.; Drera, G.; Sangaletti, L.; Malavasi, L. Enhanced Air-Stability of Sn-Based Hybrid Perovskites Induced by Dimethylammonium (DMA): Synthesis, Characterization, Aging and Hydrogen Photogeneration of the MA1-XDMAxSnBr3 System. *J Mater Chem C Mater* 2019, *7* (23), 7020–7026. https://doi.org/10.1039/c9tc01743g.

(17) Ju, D.; Zheng, X.; Liu, J.; Chen, Y.; Zhang, J.; Cao, B.; Xiao, H.; Mohammed, O. F.; Bakr, O. M.; Tao, X. Reversible Band Gap Narrowing of Sn-Based Hybrid Perovskite Single Crystal with Excellent Phase Stability. *Angewandte Chemie* 2018, *130* (45), 15084–15088. https://doi.org/10.1002/ange.201810481.

(18) Speltini, A.; Romani, L.; Dondi, D.; Malavasi, L.; Profumo, A. Carbon Nitride-Perovskite Composites: Evaluation and Optimization of Photocatalytic Hydrogen Evolution in Saccharides Aqueous Solution. *Catalysts* 2020, *10* (11), 1–11. https://doi.org/10.3390/catal10111259.

(19) Jiang, Y.; Li, K.; Wu, X.; Zhu, M.; Zhang, H.; Zhang, K.; Wang, Y.; Loh, K. P.; Shi, Y.; Xu, Q. H. In Situ Synthesis of Lead-Free Halide Perovskite Cs2AgBiBr6Supported on Nitrogen-

Doped Carbon for Efficient Hydrogen Evolution in Aqueous HBr Solution. *ACS Appl Mater Interfaces* 2021, *13* (8), 10037–10046. https://doi.org/10.1021/acsami.0c21588.

(20) Chen, G.; Wang, P.; Wu, Y.; Zhang, Q.; Wu, Q.; Wang, Z.; Zheng, Z.; Liu, Y.; Dai, Y.; Huang, B. Lead-Free Halide Perovskite Cs3Bi2xSb2–2xI9 (x ≈ 0.3) Possessing the Photocatalytic Activity for Hydrogen Evolution Comparable to That of (CH3NH3)PbI3. *Advanced Materials* 2020, *32* (39). https://doi.org/10.1002/adma.202001344.

(21) Wang, T.; Yue, D.; Li, X.; Zhao, Y. Lead-Free Double Perovskite Cs2AgBiBr6/RGO Composite for Efficient Visible Light Photocatalytic H2 Evolution. *Appl Catal B* 2020, *268*. https://doi.org/10.1016/j.apcatb.2019.118399.

(22) Guo, Y.; Liu, G.; Li, Z.; Lou, Y.; Chen, J.; Zhao, Y. Stable Lead-Free (CH3NH3)3Bi2I9 Perovskite for Photocatalytic Hydrogen Generation. *ACS Sustain Chem Eng* 2019, *7* (17), 15080–15085. https://doi.org/10.1021/acssuschemeng.9b03761.
